# Physiological and biochemical alterations in soybean by banana peel biochar under different degrees of salt stress

**DOI:** 10.1038/s41598-025-98701-w

**Published:** 2025-08-20

**Authors:** Ghulam Murtaza, Muhammad Rizwan, Muhammad Usman, Zeeshan Ahmed, Javed Iqbal, Shabir Ahmad, Mona S. Alwahibi, Humaira Rizwana, Rashid Iqbal, Gang Deng, Maximilian Lackner

**Affiliations:** 1 School of Agriculture, Yunnan University, Kunming, 650504 Yunnan China; 2 School of Ecology and Environmental Sciences, Biocontrol Engineering Research Center of Crop Diseases & Pests, Yunnan University, Kunming, 650500 Yunnan PR China; 3 Key Laboratory of Water-Sediment Sciences and Water Disaster Prevention of Hunan Province, School of Hydraulic and Environmental Engineering, Changsha University of Science & Technology, Changsha, 410114 China; 4 School of Agriculture and Biology, Shanghai Jiao Tong University, Shanghai, China; 5 Xinjiang Institute of Ecology and Geography, Chinese Academy of Sciences, Urumqi, 830011 Xinjiang China; 6 Xinjiang Institute of Ecology and Geography, Cele National Station of Observation and Research for Desert-Grassland Ecosystems, Chinese Academy of Sciences, Xinjiang, 848300 China; 7 College of Life Science, Shenyang Normal University, Shenyang, 110034 China; 8 Department of Botany, Bacha Khan University, Charsadda, 24420 Khyber Pakhtunkhwa Pakistan; 9 Department of Plant Sciences, Quaid-i-Azam University Islamabad, Islamabad, 45320 Pakistan; 10 Department of Botany and Microbiology, College of Science, King Saud University, Riyadh, 11451 Saudi Arabia; 11 Department of Agronomy, Faculty of Agriculture and Environment, The Islamia University of Bahawalpur, Bahawalpur, 63100 Pakistan; 12 Department of Life Sciences, Western Caspian University, Baku, Azerbaijan; 13 Department of Industrial Engineering, University of Applied Sciences Technikum Wien, Hoechstaedtplatz 6, Vienna, 1200 Austria

**Keywords:** Salinity stress, Biochar, Soybean, Malondialdehyde, Polyphenol oxidase, Plant sciences, Environmental sciences

## Abstract

Salt influences cellular membranes by the excessive production of reactive oxygen species, while osmolytes play a vital role in protecting plants from oxidative stress caused by salt. Biochar may alleviate the effects of salinity-induced stress on crops. The study investigated the impact of biochar supplementation on osmolyte modifications and antioxidant activity in soybean (*Glycine max* cv. AARI) under salt stress conditions. Soybean plants were exposed to 3 salinity levels (without salinity, 5, and 10 dSm^− 1^ NaCl), and different levels of biochar (without biochar, 12.5%, and 25% w/w). Root and shoot dry weight were reduced by 17% and 21%, respectively, under both salt-induced stress regimens. Salinity elevated the activities of superoxide dismutase (SOD), polyphenol oxidase (PPO), peroxidase (POD), ascorbate peroxidase (APX), and catalase (CAT) as well as O_2_•^−^ (oxygen radicals), MDA (malondialdehyde), and H_2_O_2_ (hydrogen peroxide) levels by 3.1-fold, 1.8-fold, 3.1-fold, 2.8-fold, 4.4-fold, 1.4-fold, 2.2-fold, and 2.3-fold in plants relative to control group. Furthermore, higher concentrations of soluble protein, soluble carbohydrates, glycine betaine, and proline were more pronounced at 10 dSm^− 1^ than at 5 dSm^− 1^. In contrast, incorporating biochar into soil enhanced both root and shoots dry weight by 47% and 53% respectively, compared to the absence of biochar application. Furthermore, the antioxidant levels in soybean seedlings cultivated in soil treated with biochar, particularly at a concentration of 25% biochar, decreased. Adding biochar led to a notable decrease in H_2_O_2_ (27%), O_2_^•−^(19%), and MDA (22%) concentrations, along with a reduction in the accumulation of osmotic substances in both roots and leaves. The findings demonstrate that the incorporation of biochar can safeguard soybean seedlings from NaCl-induced stress by alleviating oxidative damage.

**Supplementary Information:**

The online version contains supplementary material available at 10.1038/s41598-025-98701-w.

## Introduction

Salt-induced stress occurs due to excessive buildup of soluble salts in the soil^1^, which inhibits crop growth and production through adversely affecting osmotic balance and ion homeostasis^2^. Osmotic and ionic factors, salt-induced stress, similar to other abiotic stressors induce oxidative stress by elevating reactive oxygen species (ROS)^3,4^. ROS accumulation significantly contribute to a decline in global crop production since they influence various physiological processes by damaging oxidized proteins, inducing lipid peroxidation, and affecting nucleic acids^5,6^. Plants possess efficient methods for scavenging ROS that protect against detrimental oxidative stresses^7^. Antioxidative enzymes are the most vital elements of the defence systems. ROS accumulation due to stress is mitigated by enzymatic antioxidants, including catalase (CAT), polyphenol oxidase (PPO), peroxidase (POX), ascorbate peroxidase (APX), and superoxide dismutase (SOD)^7^. SOD metabolizes O_2_•^−^ into H_2_O_2_, thereby safeguarding cells from liability. POX, APX, and CAT facilitate the subsequent decomposition of hydrogen peroxide into oxygen and water^8^. Increased levels of antioxidants in crops have been shown to improve resistance to oxidative stress^9^.The capacity of plant cells to osmotically adjust and accumulate organic solutes is a key feature in salinity tolerance mechanisms. The buildup of key osmolytes, such as glycine betaine (N, N,N-dimethylglycine betaine), free proline, soluble proteins, and sugars in crop tissues, can signify the degree of resistance to stress achieved by osmoregulation^10^. Proline is a significant non-enzymatic antioxidant essential for microorganisms, plants, and animals to mitigate the negative impacts of ROS^11^. Mushtaq et al.^12^ reported soluble sugar accumulation in crops under saline conditions. Osmotic solutes facilitate osmotic equilibrium, regulate water-influx, and sustain turgor pressure, and it is widely recognized that crops, in reaction to saline conditions, preserve their turgor through osmotic adjustments^13,14^.

Biochar, a product of the thermal decomposition of organic materials in an oxygen-deficient environment, is differentiated from conventional charcoal by its use as a soil amendment^15,16^. Irrespective of environmental advantages, biochar significantly influences soil’s physicochemical characteristics and quality levels^17^. Moreover, studies have shown that the incorporation of biochar can enhance plant development via both direct and indirect processes^18^. The direct promotion of growth with biochar treatment relates to the provision of mineral nutrients to plants, whereas the indirect mechanism involves the improvement of the soil’s biological and physicochemical qualities^19,20^. Substantial evidence from both controlled and field experiments indicates that biochar application improves plant yield^21^. Ahmad et al.^22^ utilized biochar sourced from manure compost, which mitigated salt-induced stress; nevertheless, the processes involved remained unclear. Rivera-Solis et al.^23^ indicated that the biochar treatment to saline soil could decrease saline stress in *Solanum tuberosum* (potato) mostly because of its significant salt adsorption capacity. To date, no research has been conducted on the impact of biochar addition on osmolytes and antioxidant activities of *Glycine max* (soybean) seedlings subjected to salt-induced stress. This research was carried out to assess the impact of biochar produced from banana peel on antioxidant enzymes levels (such as SOD, PPO, POD, APX, and CAT) and O_2_•^−^, H_2_O_2_, and MDA as well as soluble protein, soluble sugars, glycine betaine, and proline levels in soybean subjected to salt stress.

## Materials and methods

### Biochar Preparation

To produce banana peel biochar, waste feedstock was collected, sun-dried under dust-free conditions, and then oven-dried at 65 °C in an air-circulating oven until a consistent weight was achieved. The desiccated substance was pulverized into fragments of 5–10 mm and then subjected to pyrolysis in a muffle furnace at 450 °C. The furnace temperature was incrementally raised from room temperature at a rate of 8 to 9 °C per minute. A residence time of 20 min was established at reaching 450 °C. Following cooling to room temperature, biochar was extracted from the furnace and further processed to a particle size of ≤ 2 mm. The biochar preparation approach follows a modified method^24^. The concentrations of C, H, N and O in biochar were quantified by an elemental analyzer (EMA 502). The basic characteristics of biochar are presented in Table S1.

### Experimental design

The trial was performed in the greenhouse at the Agricultural Research Centre, Islamia University of Bahawalpur, Punjab, Pakistan, in 2023, utilizing factorial design randomized complete block design with four replications. The experimental site is situated at coordinates 29.3981° N and 71.6908° E. Bahawalpur lies on 117 m above sea level. The climate here is dry. During the year, there is virtually no rainfall in Bahawalpur. The average annual temperature is 25.7 °C in Bahawalpur. The rainfall here is around 143 mm per year. Permissions were obtained to collect soybean (*Glycine max* cv. AARI) seeds from the Regional Agricultural Research Institute (RARI) before starting the research. Soybean plants were tested under 3 salinity levels (without salinity, 5dSm^− 1^ of NaCl, and 10 dSm^− 1^ of NaCl, which can be considered moderately and strongly saline, 0.01 M NaCl ≈ 1.3 dS m⁻¹) and 3 levels of biochar application (without biochar, 12.5%, and 25% of w/w). Salinity levels were established based on the salt tolerance range of soybean plants^25^ and biochar concentrations were determined to assess physiological parameters of soybean seedlings to varying concentrations of this organic matter (OM). Each plastic pot, measuring 20 cm in height and 8 cm in radius, contained 3 kg of soil and was planted with six soybean seeds. Before potting, the soil was thoroughly mixed with biochar and sieved via 2 mm mesh. The experimental soil’s physiochemical parameters are detailed in Table S1. Plants were cultivated in a greenhouse with regulated conditions of 60–70% relative humidity and a day/night temperature of 28 °C/21°C. Plants were watered daily using tap water throughout emergence as well as seedling growth phases to maintain the soil-water level around field capacity. Following the appearance of initial trifoliate leaves, salt was incorporated into the irrigation water provided for salt application.

### Plant biomass

Four weeks after planting, the dry weights of roots and shoots were assessed. All seedling samples’ dry weights were determined after being oven-dried at 65 °C for three days.

### Quantification of soluble protein, soluble sugars, glycine Betaine, and proline levels

The plant sample (0.5 g) was mixed in sulfosalicylic acid (5mL), and then the extract (2 mL) was put into a plastic tube, to which acetic acid (2 mL) and ninhydrin (2 mL) were mixed. The resultant mixture was heated at 105 °C for 1 h in a vessel filled with water. The mixture was extracted using toluene, and the toluene fraction absorbance obtained from the upper liquid phase was measured at 520 nm with a spectrophotometer (DR3900 UV/Vis, Hach, USA). The level of proline was quantified using a calibration curve and denoted as mg g^− 1^ DW (dry weight). The level of glycine betaine (N, N, N-dimethylglycine betaine) was quantified using the method of Gibon et al.^26^ and expressed as mg g^− 1^ FW (fresh weight). An extract of plant samples were created in test tubes (20 mL) via finely chopping leaves (0.5 g) in 0.05% toluene-water (5 mL) mixture. Test tubes were agitated for 20 h at 27 °C. Following filtration, the extract (0.5 mL) was mixed with 2 N hydrochloric acid (1 mL) solution. Then, potassium triiodide (0.1 mL) solution (composed of 10 g KI and 7.5 g I_2_ in N-ethyl-pentedrone; 100 mL) was introduced and agitated in an ice-cold water bath for 2 h. After shaking, 2 mL of ice cooled water and 10 mL of 1, 2-dichloroethane were added. The upper water layer was removed, and in the organic layer, the optical density was measured at 365 nm. Soluble sugar levels were determined using the phenol-sulfuric acid technique^27^. Glucose served as standard. The soluble sugar concentration was quantified in mg g^− 1^ FW utilizing a calibration curve. The soluble protein concentrations were determined by the Sheen technique^28^. 1 gram plant samples were added to sodium phosphate buffer (4 mL) and subsequently centrifugated at 4 °C. Dye and supernatants were pipetted into a spectrophotometer cuvette, and the absorbance was determined with a spectrophotometer at 595 nm.

### Antioxidant enzymes assessment

The SOD level is characterized as enzyme quantity that induces 50% of maximal nitroblue tetrazolium (NBT) inhibition decrease, with enzyme activity represented as Ug^− 1^ FW. The POD assay was examined via determining absorbance changes at 470 nm, with the activity being quantified as Ug^− 1^FW^29^. The catalase (CAT) activity was assessed by measuring absorbance changes at 240 nm (Ug^− 1^FW)^30^. Ascorbate peroxidase (APX) activity quantified thru observing reduction in the absorbance at 290 nm, reported as µg^− 1^ FW^31^. Polyphenol oxidase (PPO) level was assessed with the process established by Esmaeili et al.^32^and quantified as U g^− 1^ FW min^− 1^.

### Quantification of O_2•_^−^, H_2_O_2_, and MDA

To ascertain Malondialdehyde (MDA) concentrations (mmol g^− 1^ FW), 0.3 g plant specimens were homogenized in trichloroacetic acid (5%; 5 mL). The homogenate underwent centrifugation at 2000 g for 15 min at 27 °C. The supernatant was combined with 2-TBA (2-thiobarbituric acid), and heated to 85 °C for 15 min, and then cooled. Following centrifugation at 2000 x g for 15 min, absorbance was recorded at 532 nm^33^. The concentration of H_2_O_2_ was assessed with the technique established by Patterson et al.^34^. A plant sample (1 g) was homogenized in 0.1% TCA (5 mL) and centrifuged at 12000 g for 20 min. The supernatant (0.5 mL) was subsequently combined with KI (1 mL) and potassium phosphate buffer (0.5 mL). The absorbance was recorded at 390 nm. The formation of O_2_•^−^ was quantified as referenced in Yan et al.^35^.

### Statistical analyses

Variance analyses of data sets were conducted with MSTAT-C, a statistics software package; means were compared employing the LSD test at *p* ≤ 0.05. Graphs were created using Microsoft Excel.

### Statement on guidelines

All experimental studies and experimental materials involved in this research are in full compliance with relevant institutional, national and international guidelines and legislation.

## Results

### Soybean plants performance

**Fig. 1:**
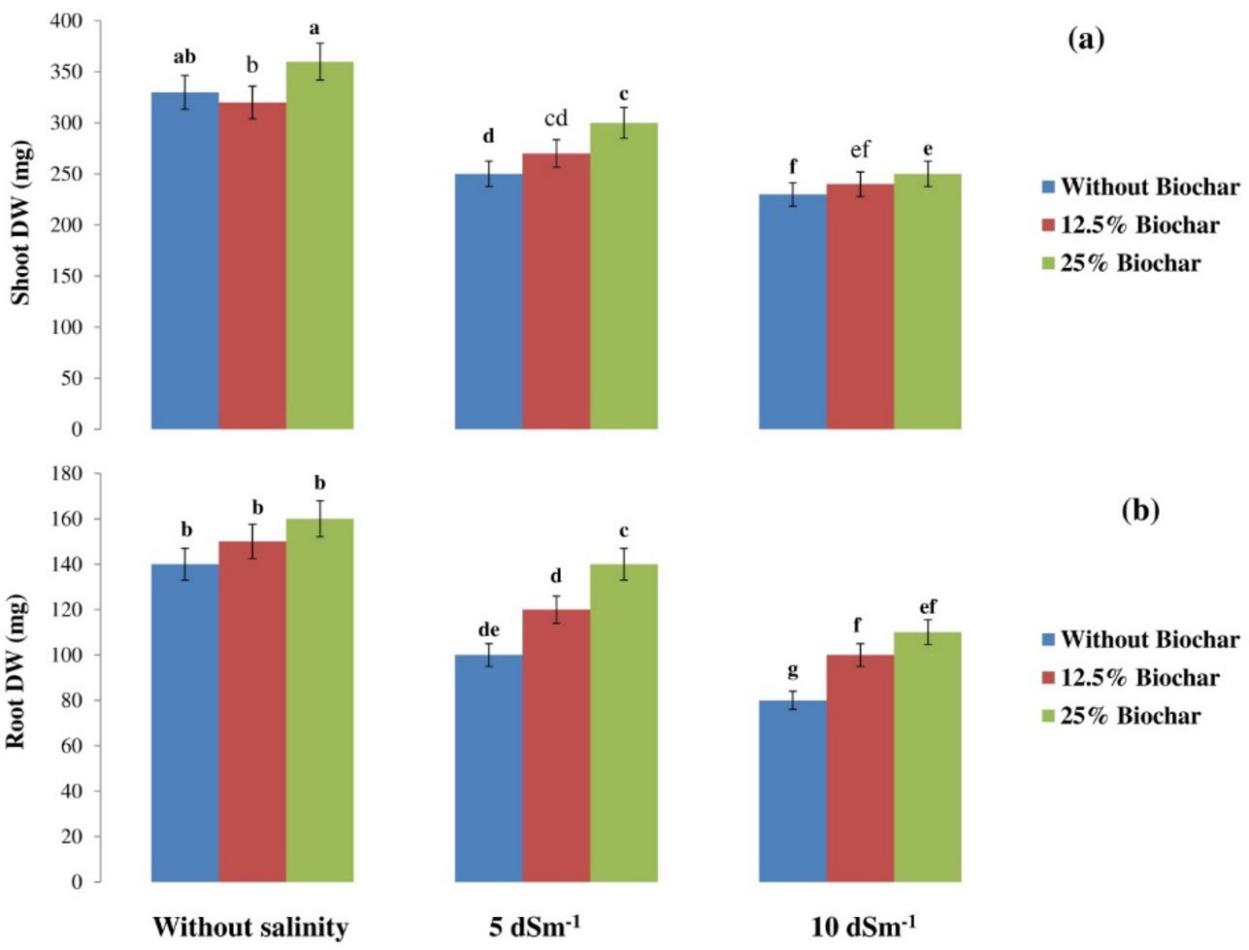
Impacts of biochar and salt levels on (**a**) shoot DW (**b**) roots DW in soybean. Bars holding same letters are not statistically different at (*P* < 0.05) based on LSD test.

As saline levels increased, root and shoot dry weight reduced with roots being more adversely affected compared to shoots (Fig. 1). Irrespective of salinity, biochar application enhanced both root and shoot dry weight (Fig. 1). Biochar with 25% of the total mass exhibited the greatest values of these metrics compared to other biochar applications under conditions without salinity (Fig. 1).

### Soluble protein, soluble sugars, glycine Betaine, and proline levels

**Fig. 2:**
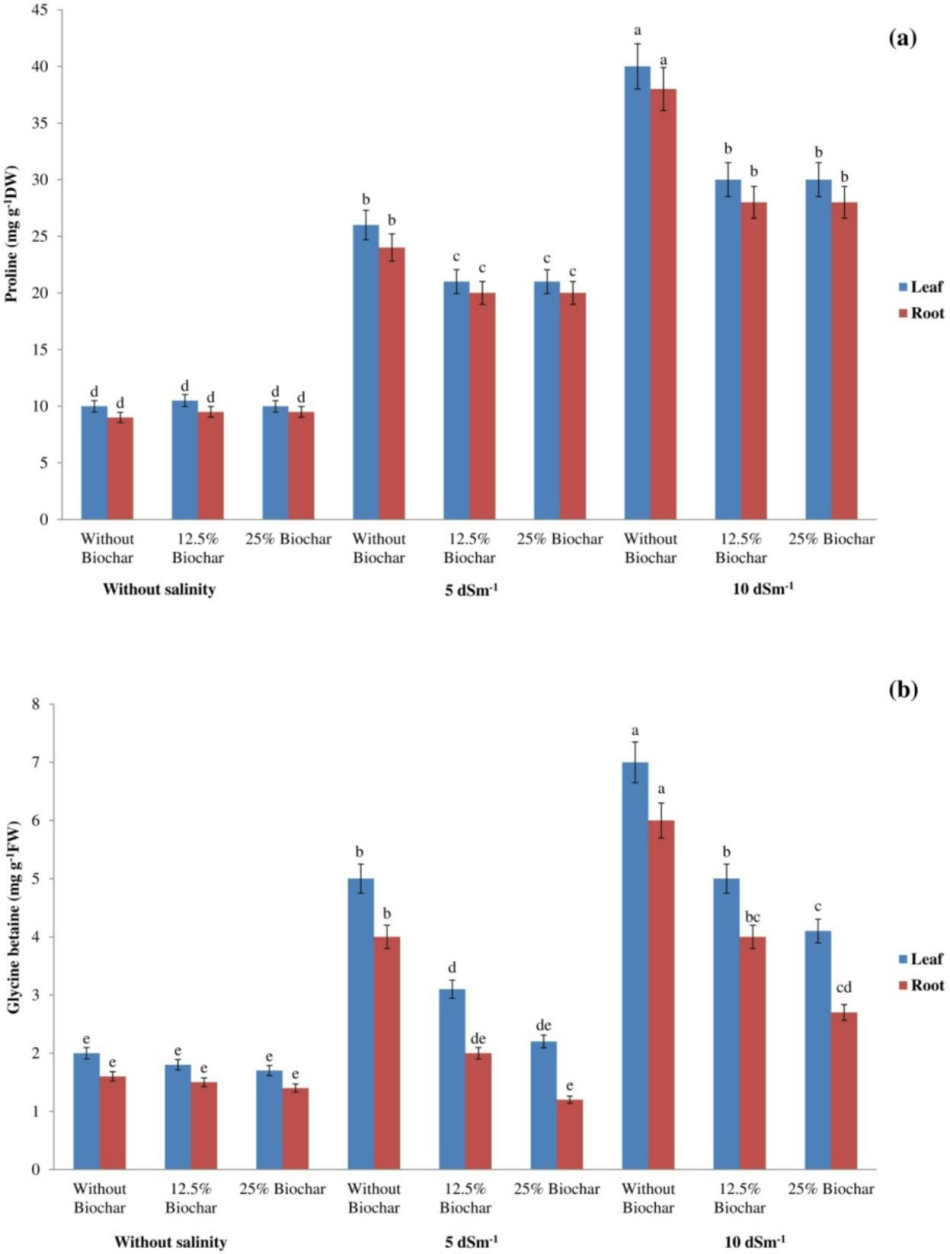

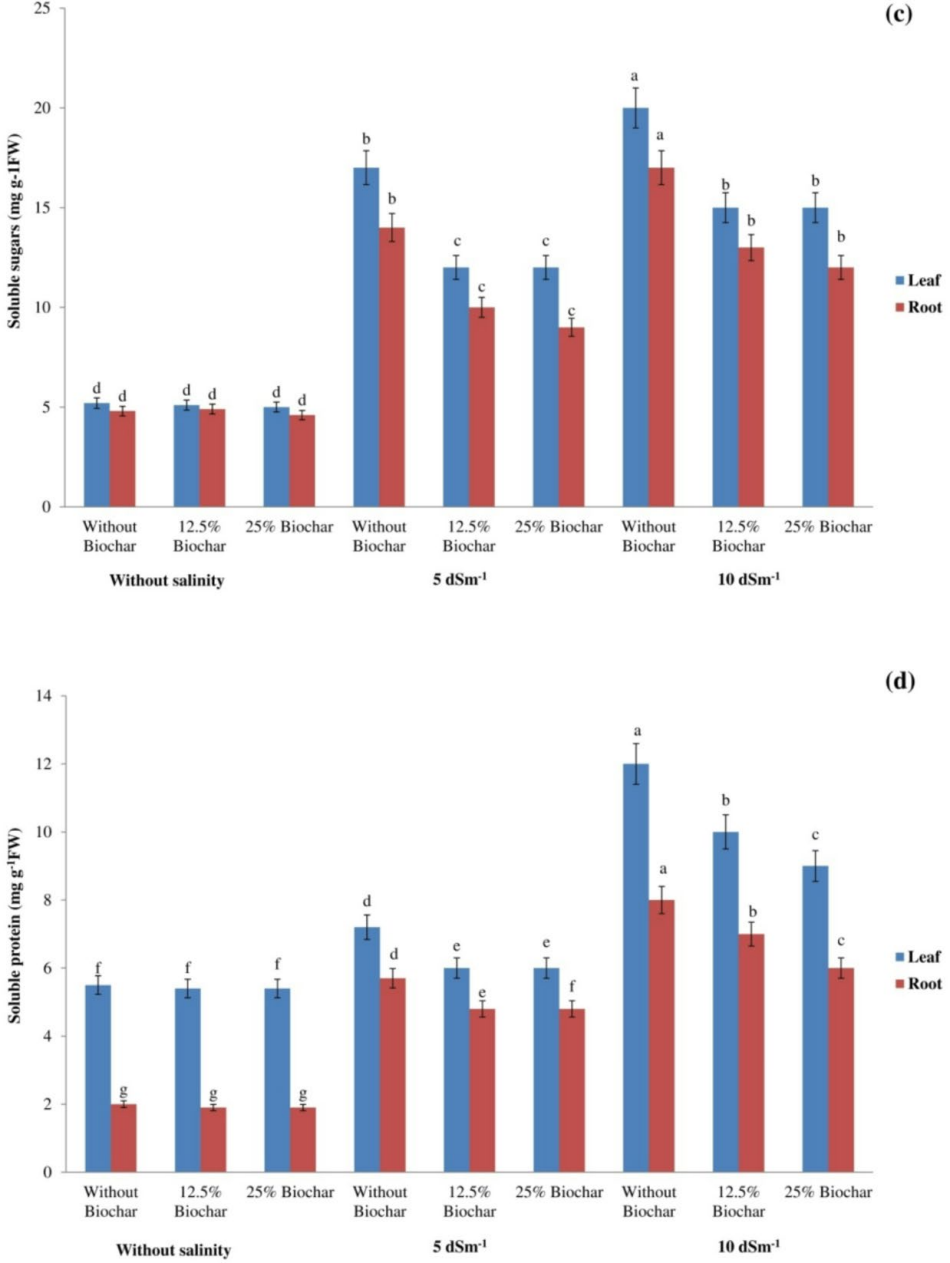
Impact of biochar and salt levels on (**a**) proline (**b**) glycine betaine (**c**) soluble sugar (**d**) soluble protein concentrations in soybean. Bars sharing the same letter are not statistically different at (*P* < 0.05) based on LSD test.

The quantification of soluble protein, soluble carbohydrates, glycine betaine, and proline in roots and leaves under salinity stress was conducted spectrophotometrically to assess their functions as osmoregulatory agents. Proline levels were found to be elevated in roots and leaves under saline conditions compared to the non-saline environment (Fig. 2). Nonetheless, the biochar addition diminished proline levels. The maximum proline concentration was 40.2 mg g^− 1^ DW in leaves and 39.3 mg g^− 1^ DW in roots, both resulting from 10 dS m^− 1^ treatment without biochar.

Saline concentrations of 5 and 10 dSm^− 1^ NaCl produced a 0.8-fold and 2-fold enhancement in root growth and a 1.5-fold and 2-fold enhancement in leaf growth, compared to non-saline conditions. In contrast, the glycine betaine concentration decreased with the addition of biochar compared to the treatment without biochar. However, the incorporation of biochar did not lead to a notable change in glycine betaine concentrations in non-saline conditions (Fig. 2). Salinity stress enhanced soluble sugar concentration in soybean roots and leaves. The concentration of soluble sugars significantly rose following the treatment with 10 dSm^− 1^NaCl (Fig. 2). Moreover, biochar treatment to soil resulted in a considerable decline in soluble sugar concentration (Fig. 2). The highest soluble sugar concentration was 19.9 mg g^− 1^ FW in leaves and 16.8 mg g^− 1^ FW in roots, both linked to the absence of biochar application at 10 dSm^− 1^ NaCl (Fig. 2). Figure 2 demonstrates that there were no significant changes in soluble sugar concentrations in the roots and leaves when biochar was applied in non-saline conditions. The concentration of soluble protein in saline-stressed plants surpassed that of the control group. Notwithstanding the salt stress, the application of biochar to growth media resulted in reduced levels of soluble protein (Fig. 2). The application of biochar did not influence the concentrations of soluble protein in roots and leaves in non-saline conditions. The highest concentration of soluble protein in roots and leaves was seen at 10 dSm^− 1^ without biochar (Fig. 2). On average, the accumulation of these osmo-regulators in leaves was greater than in roots.

### Activities of antioxidant enzymes

We examined changes in the levels of antioxidant enzymes, including CAT, PPO, POD, APX, and SOD, in response to salt stress. The results indicate that increased salt concentrations led to a rise in SOD levels (Table 1). The addition of biochar did not affect SOD activity under non-saline circumstances; however, it decreased SOD levels at 5 dSm^− 1^ and 10 dSm^− 1^ compared to the absence of biochar (Table 1). No notable variations were seen between the two biochar levels concerning SOD levels in both roots and leaves at 5 dSm^− 1^ NaCl. Table 1 presents data on the APX level of various treatments. The NaCl treatment resulted in an elevation of the APX level in both roots and leaves (Table 1). In contrast, 12.5% and 25% biochar administration exhibited significantly reduced APX levels in both roots and leaves compared to the absence of biochar (Table 1). The maximum APX activity in both the root and leaf was reported without charcoal treatment at 10 dSm^− 1^ NaCl. Moreover, APX activity showed no significant modification in non-saline conditions when banana-peel biochar was added (Table 1). The POD levels in the roots and leaves of saline-stressed soybean plants were increased relative to the control group (Table 1). Furthermore, the activity of POD was diminished with the incorporation of biochar into the soil (Table 1). Thus, the incorporation of biochar proved to be effective at salinity levels of 5 dSm^− 1^ and 10 dSm^− 1^; however, no notable differences were detected among biochar treatments under non-saline conditions. PPO activity increased with the enhancement of salt stress in roots and leaves (Table 1). Nonetheless, PPO activity diminished in both root and leaf after biochar application. The maximum PPO levels in root and leaf were 7.1 and 10.1 Ug^− 1^FW min^− 1^, respectively, seen in biochar-less experiments at 10 dSm^− 1^. Furthermore, PPO levels of soybeans exhibited no significant variation in saline-less conditions when banana-peel biochar was incorporated into the soil. The findings revealed a substantial disparity in CAT activity between salt-stressed plants and the control group. In comparison to the control, plants grown with biochar demonstrated a 2.8-fold and 4.9-fold increase in root catalase (CAT) activity, and a 2.4-fold and 4.4-fold enhancement in leaf CAT activity at salinity levels of 5 and 10 dSm^− 1^, respectively. The addition of biochar in saline conditions reduced CAT activity in both leaves and roots, while CAT activity remained constant with biochar in non-saline environments (Table 1). The greatest CAT activity in roots and leaves was seen under 10dSm^− 1^ without biochar. Antioxidant enzymes such as PPO, POD, as well as APX were higher in roots than in leaves. By contrast, the activities of CAT and SOD in leaves surpassed those in roots, suggesting a scavenging mechanism for ROS elimination in both leaves and roots.

**Table 1:** Soybean seedling antioxidant activity under varying saline levels and biochar treatment.

Salt level	Biochar dose	SOD (U g^− 1^FW)		APX (U g^− 1^FW)		POD (U g^− 1^FW)		PPO (U g^− 1^FW min^− 1^)		CAT (U g^− 1^FW)	
		Root	Leaf	Root	Leaf	Root	Leaf	Root	Leaf	Root	Leaf
Without salinity	Without biochar	0.19 ± 0.002e	0.49 ± 0.02e	0.40 ± 0.03e	0.19 ± 0.008f	0.19 ± 0.02f	0.13 ± 0.007e	1.40 ± 0.04f	0.69 ± 0.03f	0.30 ± 0.007e	0.29 ± 0.007 g
	12.5% biochar	0.24 ± 0.03e	0.44 ± 0.07e	0.50 ± 0.02e	0.29 ± 0.04f	0.25 ± 0.10f	0.13 ± 0.003e	1.20 ± 0.03f	0.50 ± 0.02f	0.15 ± 0.002e	0.45 ± 0.02 g
	25% biochar	0.16 ± 0.003e	0.50 ± 0.01e	0.26 ± 0.006f	0.33 ± 0.007f	0.14 ± 0.007f	0.14 ± 0.02e	1.04 ± 0.07f	0.59 ± 0.06f	0.40 ± 0.02e	0.19 ± 0.02 g
5 dSm^− 1^	Without biochar	1.10 ± 0.04c	2.09 ± 0.10c	4.50 ± 0.07c	3.50 ± 0.02c	5.89 ± 0.09d	3.39 ± 0.07c	5.69 ± 0.08b	3.49 ± 0.09d	1.29 ± 0.04c	1.79 ± 0.03d
	12.5% biochar	0.80 ± 0.07d	1.09 ± 0.06d	3.39 ± 0.1d	1.89 ± 0.07d	4.59 ± 0.3e	2.89 ± 0.04d	3.30 ± 0.07d	2.29 ± 0.04e	0.89 ± 0.04d	1.29 ± 0.02e
	25% biochar	0.69 ± 0.07d	1.04 ± 0.02d	3.30 ± 0.07d	1.50 ± 0.07e	4.19 ± 0.2e	2.79 ± 0.20d	2.70 ± 0.08e	2.10 ± 0.05e	0.79 ± 0.02d	1.20 ± 0.07f
10 dSm^− 1^	Without biochar	2.09 ± 0.10a	3.10 ± 0.07a	7.50 ± 0.19a	5.39 ± 0.06e	11.3 ± 0.30a	5.30 ± 0.20a	9.60 ± 0.20a	6.59 ± 0.20a	1.89 ± 0.03a	2.70 ± 0.07a
	12.5% biochar	1.29 ± 0.29b	2.29 ± 0.07b	6.19 ± 0.10b	3.79 ± 0.07b	8.49 ± 0.2b	4.10 ± 0.3b	5.80 ± 0.19b	5.19 ± 0.07b	1.49 ± 0.06b	2.30 ± 0.04b
	25% biochar	1.29 ± 0.10b	2.09 ± 0.06c	6.09 ± 0.11b	3.49 ± 0.02c	6.9 ± 0.29c	3.89 ± 0.2b	5.19 ± 0.40c	4.49 ± 0.09c	1.40 ± 0.07c	2.10 ± 0.02c

Means sharing the different letter case are significantly different at 5% probability level.

### MDA concentration, H_2_O_2_ levels, and O_2_•^−^ production

Table 2 illustrates the detrimental impact of salinity on MDA level, H_2_O_2_ level, and O_2_•^−^ formation in soybean seedlings. Root MDA levels increased significantly by 0.7-fold and 2.2-fold, while leaf MDA levels climbed by 1.6-fold and 4.1-fold at saline levels of 5 and 10 dSm^− 1^, respectively, compared to the control. The concentration of H_2_O_2_ in the roots increased by 0.9-fold and 2.3-fold, while in the leaves it increased by 1.1-fold and 1.8-fold at 5 and 10 dSm^− 1^, respectively, compared to the control. At 5 dSm^− 1^, the concentration of O_2_^•−^ in roots and leaves increased by 1.4-fold and 0.8-fold, respectively, while at 10 dSm^− 1^, it rose by 2.6-fold and 1.9-fold. Conversely, biochar incorporation into soil led to a reduced MDA level, H_2_O_2_ level, and O_2_•^−^ production. Furthermore, MDA, O_2_•^−^, and H_2_O_2_ levels in roots and leaves exhibited no significant variation with biochar addition in non-saline conditions. Thus, the greatest values were associated with biochar-less application at 10 dSm^− 1^.

**Table 2:** Levels of MDA, O_2_•^−^, and H_2_O_2_ of soybean seedlings under varying saline Levels and Biochar treatment.

Salt level	Biochar dose	MDA (mmol g^− 1^FW)		H_2_O_2_ (µmol g^− 1^FW)		O_2_•^−^ (µmol g^− 1^FW h^− 1^)	
		Root	Leaf	Root	Leaf	Root	Leaf
Without salinity	Without biochar	2.39 ± 0.06e	1.29 ± 0.06c	0.09 ± 0.002e	0.12 ± 0.006e	0.10 ± 0.007f	0.11 ± 0.007f
	12.5% biochar	2.20 ± 0.02e	1.19 ± 0.06c	0.06 ± 0.003e	0.11 ± 0.002e	0.11 ± 0.007f	0.14 ± 0.005f
	25% biochar	2.34 ± 0.07e	1.20 ± 0.07c	0.06 ± 0.007e	0.11 ± 0.007e	0.11 ± 0.002f	0.14 ± 0.006f
5 dSm^− 1^	Without biochar	8.29 ± 0.50c	3.19 ± 0.60b	0.19 ± 0.03bc	0.30 ± 0.03b	0.26 ± 0.02b	0.40 ± 0.03c
	12.5% biochar	4.70 ± 0.19d	1.60 ± 0.30c	0.14 ± 0.007d	0.20 ± 0.03 cd	0.17 ± 0.006d	0.30 ± 0.007d
	25% biochar	4.09 ± 0.19d	1.29 ± 0.30c	0.12 ± 0.006d	0.14 ± 0.03de	0.14 ± 0.004e	0.27 ± 0.007e
10 dSm^− 1^	Without biochar	13.9 ± 0.39a	5.29 ± 0.60a	0.30 ± 0.02a	0.40 ± 0.007a	0.36 ± 0.007a	0.60 ± 0.02a
	12.5% biochar	9.91 ± 0.19b	3.49 ± 0.20b	0.19 ± 0.006b	0.24 ± 0.03bc	0.26 ± 0.006b	0.39 ± 0.007b
	25% biochar	9.20 ± 0.30bc	2.79 ± 0.39b	0.17 ± 0.02c	0.20 ± 0.02 cd	0.19 ± 0.007c	0.40 ± 0.007c

Means sharing the different letter case are significantly different at 5% probability level.

## Discussion

This study found a reduction in the dry weight of roots and shoots under salt stress; however, the activities of SOD, PPO, POD, APX, and CAT were significantly elevated with the application of 5 and 10 dSm^− 1^ NaCl. The decreased growth of soybean seedlings under salt stress may be attributed to reduced sodium ion toxicity and limited water availability for the plants. Salt stress is a significant abiotic stress that inflicts considerable harm on plants by impacting morphological, molecular, and biochemical levels. It inhibits plant growth, diminishes photosynthesis, and induces oxidative damage through the generation of reactive oxygen species and the peroxidation of essential biological components^36,37^.

Our findings demonstrated that salt caused oxidative stress in perennial soybean leaves, as evidenced by elevated H_2_O_2_, MDA, and O_2_•^−^ levels. As salinity levels increased to 10 dSm^− 1^, there was an elevation in antioxidant activity, H_2_O_2_, MDA, and O_2_•^−^ levels, and osmolytic contents. Salinity-induced osmotic and ionic stress leads to excessive ROS production; ultimately causing oxidative damage to cellular organelles and membrane components, and at extreme levels, result in cell and plant mortality. The antioxidant defense system safeguards the plant from salt-induced oxidative damage by detoxifying ROS and regulating the equilibrium of ROS production during salt stress^38^. Biotic and abiotic stresses can induce ROS generation and accumulation, such as OH, H_2_O_2_, and O_2_•^−^, typically produced by several metabolic pathways^39^. ROS impact several physiological processes by depleting oxidized proteins and nucleic acids, as well as promoting lipid peroxidation^40^. Lipid peroxidation is considered the most harmful process in all living beings. Membrane damage is sometimes used as a sole criterion for evaluating the degree of lipid degradation under various stresses^41^. Lipid peroxidation occurs when ROS exceed a certain threshold, directly disturbing normal cellular activities and impairing oxidative damage via producing lipid-based radicals^42,43^. Conversely, enzymatic antioxidant mechanisms comprising various scavengers, including SOD, POD, APX and CAT are essential enzymes for ROS scavenging in the plants^44^. SOD catalyzes O_2_•^−^ dismutation, converting one O_2_•^−^ into H_2_O_2_. Catalase (CAT) is a tetrameric, heme-containing enzyme that directly dismutates hydrogen peroxide into water and oxygen, playing a crucial role in ROS detoxification during stress^42,45^. APX is considered to have a crucial function in scavenging reactive oxygen species, particularly hydrogen peroxide, and safeguarding cells in the higher species. POD is primarily situated in vacuoles and the apoplastic space, where it significantly catalyzes hydrogen peroxide conversion to water and O_2_•^[− 46^. Elevated activity of PPO during stress signifies its capacity to oxidize and decompose hazardous chemicals, including phenolic substances, which are often known to accumulate under salinity^47^. Results indicate that CAT, PPO and SOD levels in leaves surpassed those in roots; conversely, POD and APX levels were elevated in roots relative to leaves. The levels of hydrogen peroxide and O_2_•^−^ in roots were lower than in leaves. This effective scavenging system may exist since the roots are initial plant organs to encounter stresses and contaminants^48,49^.

The findings of our research indicate that levels of soluble proteins, soluble sugars, glycine betaine, and proline in plants exposed to salinity were higher than in non-stressed plants. In reaction to salinity stress, many plants produce glycine betaine to mitigate the detrimental effects of salt stress and preserve cellular osmotic balance. Glycine betaine is extensively researched by altering its metabolic pathways via transgenic methods. The betaine aldehyde dehydrogenase gene from the halophyte *Suaeda liaotungensis*, which encodes an enzyme that converts betaine aldehyde to betaine, is overexpressed in transgenic tobacco plants, resulting in markedly enhanced salt tolerance^50^. Osmotic adaptation is a crucial mechanism that allows plants to endure salinity stress^51^. A common response to salt stress is the decrease in cell water potential due to increased solute concentrations or osmotic adjustment, which is essential for maintaining cell water content and turgor pressure^52^. Glycine betaine recognized as an efficient osmoprotectant that accumulates in specific species under stress conditions. It functions a crucial role as non-osmotic modulator that preserves the integrity of cell membranes^53^. The variation in soluble sugars concentrations in canola under salinity^54^. The increase in soluble sugar levels with elevated salt concentrations may be attributed to the accumulation of soluble sugars and starch in plants under stress. A low concentration of free amino acids and proline accumulation in the cytoplasm can swiftly reach elevated levels and enhance the osmotic potential of the vacuole more efficiently. Amino acids serve as potential osmoprotective solutes, diminishing osmotic potential in specific tissues under stress^55^. On average, the concentrations of soluble proteins, soluble sugars, and glycine betaine in the leaves of soybean seedlings were higher than the roots.

In our study, the utilization of biochar increased the dry weight of both shoots and roots in soybean seedlings, while simultaneously reducing the levels of antioxidant activities, organic osmolytes, and concentrations of MDA, O_2_•^−^, and hydrogen peroxide in the leaves and roots under salinity stress. It was due to the biochar can augment soybean yield by improving soil nutrient levels, including the availability of phosphorus and certain trace elements, and raising the plant’s accumulation and absorption of potassium, phosphorus, and nitrogen^56^. Research indicates that the incorporation of biochar into soil can augment crop yields by enhancing biological and physiochemical characteristics of soil, the application of biochar improves soil physical and chemical characteristics, stimulates enzymatic activity, accelerates soil respiration, and fosters microbial biomass development^57,58^. The beneficial effects of biochar incorporation were attributed to the decrease in MDA, O_2_^•−^, and hydrogen peroxide levels, leading to enhanced root and shoot dry weight of soybeans in saline conditions. The positive reactions to biochar addition were more pronounced in 25% biochar than in 12.5% biochar, can be attributed to the increased biochar surface area and reactivity, enhanced nutrient availability and uptake, increased water holding capacity and aeration, enhanced microbial activity and diversity, and reduced salt stress and oxidative damage. Abrol et al.^56^ suggested that the incorporation of biochar, which is persistent in soil, may alleviate the impacts of saline stress, due reducing educing Na^+^ uptake and leads to enhanced yield and quality. Abiotic stress leads to the overproduction of reactive oxygen species in plant cells. The balance between the generation and elimination of reactive oxygen species is crucial for maintaining cellular metabolic functions under stress, therefore mitigating oxidative damage. The antioxidant system may modulate the levels of reactive oxygen species in plant tissues^59^. Salt stress adversely affects plants at all growth stage, leading to plant mortality and diminished yield. Elevated salinity influences plant growth by altering physiological processes^60^. Plants have established an antioxidant defence mechanism to mitigate oxidative damage caused by salt stress. The current study revealed that salinity stress elevated the activities of CAT, POD, and SOD in comparison to non-saline conditions. Salinity stress elevates the activities of CAT, POD, and SOD by activating the antioxidant defense system, which involves transcriptional and post-translational activation of antioxidant enzymes. The mechanisms of elevated CAT, POD, and SOD activities involve the conversion of ROS into less reactive species, reducing oxidative damage and promoting cellular homeostasis. The elevation of antioxidant enzymes may result from the activation of plant defence systems^61^. The application of biochar can modulate the production of antioxidant enzymes in plants, hence enhancing their resilience to salt stress^62^. Several studies have demonstrated the impact of biochar on oxidative stress and antioxidant enzyme activity in plants subjected to salt stress. Biochar may enhance plant development under salt stress by mitigating oxidative damage. Previous research indicates that biochar treatment reduced antioxidant enzyme activity in maize subjected to salt stress^63^. Our findings indicate that soybean seedlings in soil enriched with 25% biochar showed a decrease in antioxidant enzymes, including SOD, PPO, POD, APX, and CAT, under salinity stress. The decrease in antioxidant enzymes, including SOD, PPO, POD, APX, and CAT, in soybean seedlings grown in soil enriched with 25% biochar under salinity stress can be attributed to biochar-mediated mechanisms, including improved soil structure, reduced ROS generation, and enhanced nutrient availability, leading to antioxidant enzyme downregulation and redox balance maintenance. The addition of biochar significantly alleviated the decrease in dry weight of both root and shoot at 5 and 10 dSm^− 1^ NaCl by improving soil structure, increasing nutrient availability, reducing soil salinity, and promoting plant growth through reduced oxidative stress, improved water relations, and hormonal regulation. It also reduced hydrogen peroxide and O_2_^•−^ and inhibited lipid peroxidation in plant cells. Biochar reduced the concentrations of hydrogen peroxide and O_2_^•−^, both reactive oxygen species, in the leaves and roots of beans exposed to salt treatments, leading to a decrease in organic osmolytes and antioxidant activities. Thus, addition of biochar to soil mitigates the cytotoxic effects of Cl and Na by reducing hydrogen peroxide and O_2_•^−^ levels, which results in decreased antioxidant activities. Biochar exhibited a substantial residual impact on mitigating the detrimental effects of salinity. Adhikari et al.^64^ shown that biochar application diminished plant Na absorption by temporary Na^+^ binding, attributed to its high adsorption capacity, which alleviated osmotic stress by improving soil moisture content and releasing mineral nutrients into the soil solution.

## Conclusion

Elevated levels of antioxidant activity, ROS level, and osmolyte level were more significant at 10 dSm^− 1^ compared to 5 dSm^− 1^ NaCl. Compared to without-biochar administration, the application of biochar to soil improved dry weight of both shoots and roots. The biochar application, specifically at a concentration of 25%, significantly reduced hydrogen peroxide and O_2_•^−^ levels as well as osmotic adjustments, antioxidant activities and malondialdehyde content. Furthermore, we have exhibited variations in antioxidant activity responses and osmotic adaptations to biochar treatments under saline stress in soybean seedlings. Furthermore, unmeasured parameters were altered by biochar in un-stressful conditions. Biochar also reduced hydrogen peroxide and O_2_•^−^ and prevented lipid peroxidation in cells in plants. Results indicated that biochar could protect soybean seedlings from salt stress by mitigating oxidative damage. Biochar has demonstrated its ability to adsorb agricultural chemicals and nutrients, thereby mitigating leaching of these substances into ground and surface water, while also enhancing CEC in acidic soils, which further augments the soil’s nutrient retention capability. Based on these findings on biochar in soil, we advocate conducting additional experiments with various biochar concentrations under saline conditions also with additional plants to develop low-cost, environmentally friendly approaches for combating salty soil degradation.

## Electronic supplementary material

Below is the link to the electronic supplementary material.

Supplementary Material 1

## Data Availability

All the raw data in this research can be obtained from the corresponding authors upon reasonable request.

## Declarations

### Permission

Permissions or licenses were obtained to collect Soybean (Glycine max cv. AARI soybean) seeds from Regional Agricultural Research Institute (RARI) before starting the research.
