# Supplementary material for: Physiological and biochemical alterations in soybean by banana peel biochar under different degrees of salt stress
Source: Sci Rep. 2025 Aug 20;15:30532. doi: 10.1038/s41598-025-98701-w (PMC12368208; doi:10.1038/s41598-025-98701-w)
Supplement: Supplementary file 1 — Supplementary Material 1 [file 41598_2025_98701_MOESM1_ESM.docx]

**Table S1** Physicochemical property of biochar and experimental soil.

| **Soil** | | **Biochar** | |
| --- | --- | --- | --- |
| Texture | Silty-loam | O (%) | 30.11 |
| Electrical conductivity (EC) (dSm^-1^) | 1.19 | H (%) | 1.8 |
| pH | 8.2 | C (%) | 33.12 |
| Organic C (gkg^-1^) | 13.9 | N (%) | 0.80 |
| Total nitrogen (%) | 0.06 | K (mgkg^-1^) | 3199 |
| K (mgkg^-1^) | 170 | Na (mgkg^-1^) | 8.4 |
| P (mgkg^-1^) | 29 | Mg (mgkg^-1^) | 959 |
| Cation exchange capacity (CEC) (cmolkg^-1^) | 16.9 | Ca (mgkg^-1^) | 3501 |
|  |  | pH | 7.7 |
|  |  | CEC (cmolkg^-1^) | 21.4 |
